# Highly sensitive SERS platform for pathogen analysis by cyclic DNA nanostructure@AuNP tags and cascade primer exchange reaction

**DOI:** 10.1186/s12951-024-02339-1

**Published:** 2024-02-26

**Authors:** Yunju Xiao, Shihua Luo, Jiuxiang Qiu, Ye Zhang, Weijiang Liu, Yunhu Zhao, YiTong Zhu, Yangxi Deng, Mengdi Lu, Suling Liu, Yong Lin, Aiwei Huang, Wen Wang, Xuejiao Hu, Bing Gu

**Affiliations:** 1 Laboratory Medicine, Guangdong Provincial People’s Hospital (Guangdong Academy of Medical Sciences), Southern Medical University, Guangzhou, 510080 People’s Republic of China; 2 Department of Laboratory Medicine, Nanfang Hospital, Southern Medical University, Guangzhou, 510515 People’s Republic of China; 3 Center for Clinical Laboratory Diagnosis and Research, Affiliated Hospital of Youjiang Medical University for Nationalities, Baise, Guangxi 533000 People’s Republic of China; 4 Department of Laboratory Medicine, Guangzhou Eighth People’s Hospital, Guangzhou Medical University, Guangzhou, 510515 People’s Republic of China; 5 Key Laboratory of Research on Clinical Molecular Diagnosis for High Incidence Diseases in Western Guangxi of Guangxi Higher Education Institutions, Affiliated Hospital of Youjiang Medical University for Nationalities, Baise, Guangxi 533000 People’s Republic of China

**Keywords:** SERS tags, Primer exchange reaction, Bacterial detection, Early infection diagnosis

## Abstract

The capacity to identify small amounts of pathogens in real samples is extremely useful. Herein, we proposed a sensitive platform for detecting pathogens using cyclic DNA nanostructure@AuNP tags (CDNA) and a cascade primer exchange reaction (cPER). This platform employs wheat germ agglutinin-modified Fe_3_O_4_@Au magnetic nanoparticles (WMRs) to bind the *E. coli* O157:H7, and then triggers the cPER to generate branched DNA products for CDNA tag hybridization with high stability and amplified SERS signals. It can identify target pathogens as low as 1.91 CFU/mL and discriminate *E. coli* O157:H7 in complex samples such as water, milk, and serum, demonstrating comparable or greater sensitivity and accuracy than traditional qPCR. Moreover, the developed platform can detect low levels of *E. coli* O157:H7 in mouse serum, allowing the discrimination of mice with early-stage infection. Thus, this platform holds promise for food analysis and early infection diagnosis.

**Supplementary Information:**

The online version contains supplementary material available at 10.1186/s12951-024-02339-1.

## Introduction

Pathogenic microorganisms are a group of bacteria that can invade the human body and cause infections or even infectious diseases, currently, including *Escherichia coli* O157:H7, *Acinetobacter baumannii*, and *Staphylococcus aureus*, which pose a serious risk to public health [1–3]. Prompt detection of these pathogens is important for effective infection control and clinical diagnosis. The common means of detecting pathogens include culture, PCR and mass spectrometry [4–6], but all these methods face some problems. For instance, the widespread application of the conventional microbial culture identification approach is hampered by its length of time and complexity. PCR often yields false positives and non-specific amplification [7, 8]. Although mass spectrometry offers rapid results, it necessitates an expensive instruments and skilled workers, making it a challenging process [9]. Therefore, there is an urgent need for faster and more accurate methods of pathogen detection is critical.

Surface-enhanced Raman spectroscopy (SERS) is a technique that capitalizes on the surface enhancement effect to amplify Raman scattering signals of samples, enabling highly sensitive detection of low concentration samples [10–14]. This technique boasts advantages such as high selectivity, sensitivity, and non-destructiveness, making it a powerful tool for analyzing proteins and biomolecules [15]. SERS analyses are bifurcated based on the interaction between signaling molecules and active substrates into labeling methods and “label-free” methods [16]. The label-free approach allows direct sample analysis to obtain a more complete structural spectrum of the substance [17, 18], whereas the label-based method, utilizing specific Au nanoparticles (AuNPs) as Raman tags, ensures amplified and quantifiable signals, thereby exhibiting superior reproducibility and sensitivity. For instance, Duan’s group presented a label-based SERS technique for detecting traces of S. typhimurium [19–21]. The detection of *Listeria monocytogenes* has been achieved using multiple SERS tags that employ distinct Raman reporter molecules and specialized identification components [22]. Despite the remarkable performance of AuNP tags, current AuNP-based SERS tags still encounter several drawbacks including time-intensive and intricate conjugation processes of AuNP -based SERS tags, low DNA and Raman reporter molecule loading density, and the Raman reporter’s susceptibility to interference upon direct adsorption onto AuNPs [23]. These limitations underscore the pressing need for the development of more efficient and stable AuNP tags for SERS detection.

In addition to AuNP tags, an extraordinary amplification method is vital for establishing an effective and feasible SERS detection platform. The primer exchange reaction (PER) is a thermostatic nucleic acid amplification method that can rapidly synthesize long single-stranded DNA with repetitive sequences through a cascade of extension and replacement reactions mediated by a DNA replacer enzyme and a concise primer sequence [24–26]. Given its specificity and robust signal amplification, PER has been adopted for the sensitive detection of various biomarkers [27–29]. For example, An et al. used PER to achieve sensitive and convenient detection of HIV gene fragments [30]. Li et al. adopted PER cascade amplification for the detection of miRNA [31–33]. However, the use of appropriate and sensitive PER for pathogenic bacteria detection remains underexplored, indicating a research gap that warrants investigation.

This study therefore developed a rapid and sensitive SERS-based biosensor for the detection of *E.coli* O157:H7 by using cyclic DNA nanostructure@AuNP tags (CDNA) and a cascade primer exchange reaction (cPER). The wheat germ agglutinin-modified raspberry-like Fe_3_O_4_@Au (WGA-MRs, WMRs), which bind to the cell wall molecules of *E. coli* O157:H7 by highly expressed N-acetyl-D-glucosamine on WMRs, was utilized to capture the target efficiently [34, 35]. DNA aptamer is a special functional nucleic acid sequence, which is screened by SELEX technique [36]. The aptamer forms an antibody-like spatial conformation through base stacking force or hydrogen bonding force, thus achieving affinity connection with the target protein [37]. For example, Wu et al. reported an aptamer E18R-42, which can label the *E. coli* O157:H7 [38, 39]. The E18R-42 was employed to form Primer-Aptamer, which the aptamer link with the protein on the cell wall in this work. The branched DNA products constructed by the cascade PER were used to bind the CDNA tags in the presence of target pathogens. These CDNA tags were synthesized by in situ assembly on the surface of AuNPs via the hybridization chain reaction of three DNA sequences (La, Lb, and Lc), and Raman reporter molecules (methylene blue, MB) were incorporated within the cyclic DNA nanostructure to amplify and stabilize the SERS signal. This SERS platform combines the advantages of CDNA tags with the cascade PER method to detect trace pathogens, proposing a reliable sensing platform for the early and accurate detection of infectious diseases.

## Results and discussion

### Principle of the CDNA-based SERS platform

**Fig. 1:**
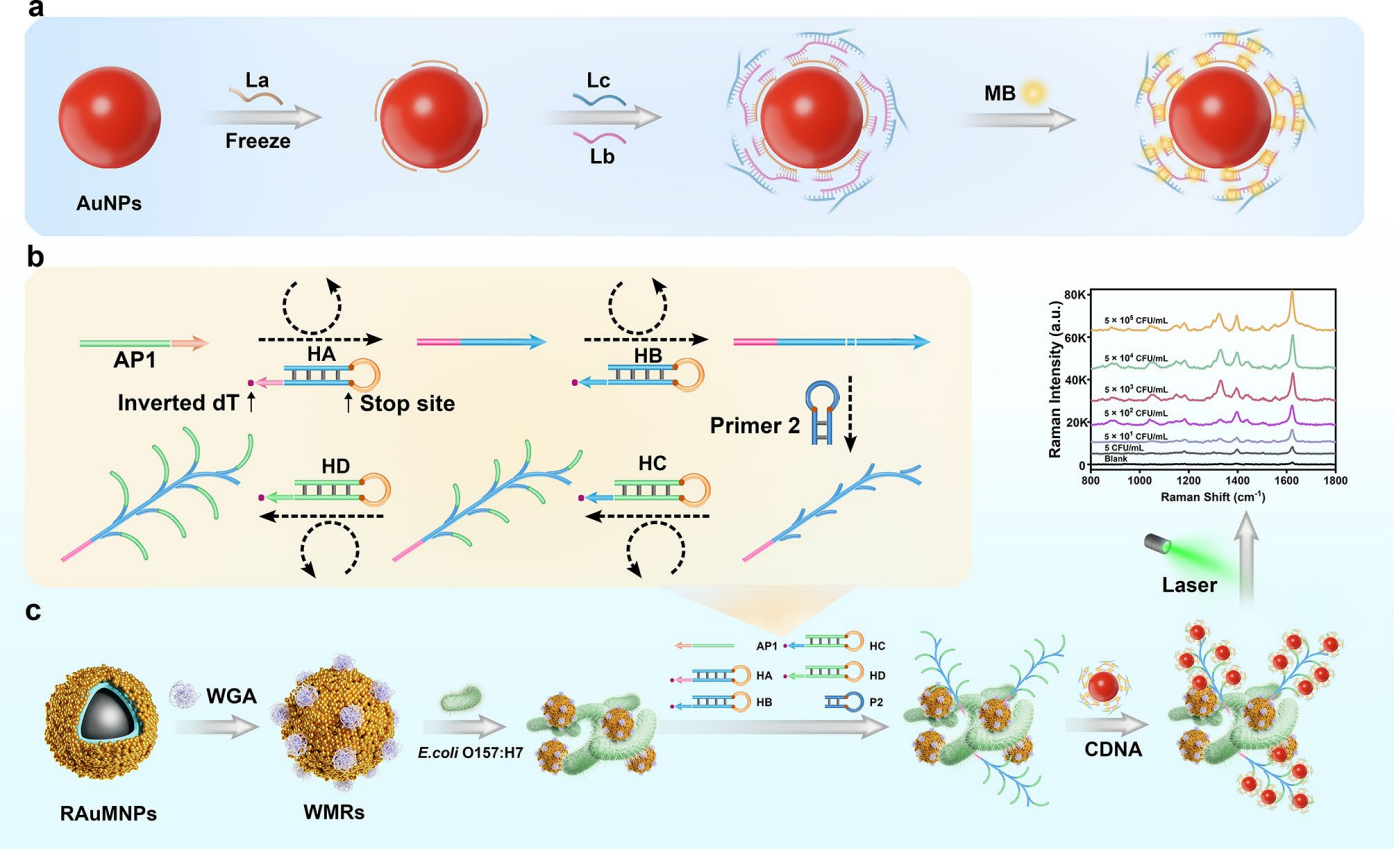
**a** Schematic illustration of the fabrication of cyclic DNA nanostructure@AuNP tags (CDNA); **b** Principle of Aptamer-activated cascade PER for signal amplification of *E. coli* O157:H7; **c** The principle of target bacteria detection using a CDNA-based SERS platform

Figure 1 elucidates the underlying principle of the method developed in this study. The conjugated DNA (cDNA) assembly was performed in situ on gold nanoparticles (AuNPs). Initially, the DNA strand labeled La was anchored onto the AuNP surface via gold-thiolate bonds. This was succeeded by the sequential conjugation of DNA strands Lb and Lc to La, creating a circular DNA nanostructure that encased the AuNP, thus providing a scaffold for subsequent hybridization events with SERS reporter molecules and the target sequence (Fig. 1a).

WMRs were synthesized and employed for the selective capture of E. coli O157:H7 (Fig. 1b and c). The cascade primer exchange reaction (cPER), constituting DNA polymerase, Aptamer-primer 1, primer 2, and a series of hairpins (HA, HB, HC, and HD), was initiated following the WMR-mediated capture of the target bacteria. The binding of Aptamer-primer 1 to E. coli O157:H7 facilitated the formation of the Aptamer-primer 1/E. coli O157:H7/WMRs complexes, triggering the cPER.

The cPER began with the hybridization of hairpin primer HA to the complementary regions of Aptamer-primer 1, instigating a templated synthesis of a new sequence at the primer’s 3’ terminus. This newly synthesized sequence engaged in further hybridization with hairpins HB and HC. Subsequently, HC directed the elongation of primer 2, leading to the iterative extension of hairpin D and the synthesis of branched DNA structures. These structures were then available for the hybridization of multiple cDNA tags, forming a multitude of SERS-active hot spots, resulting in a pronounced enhancement of the SERS signal, (Fig. 1c).

### Characterization of the CDNA tags

**Fig. 2:**
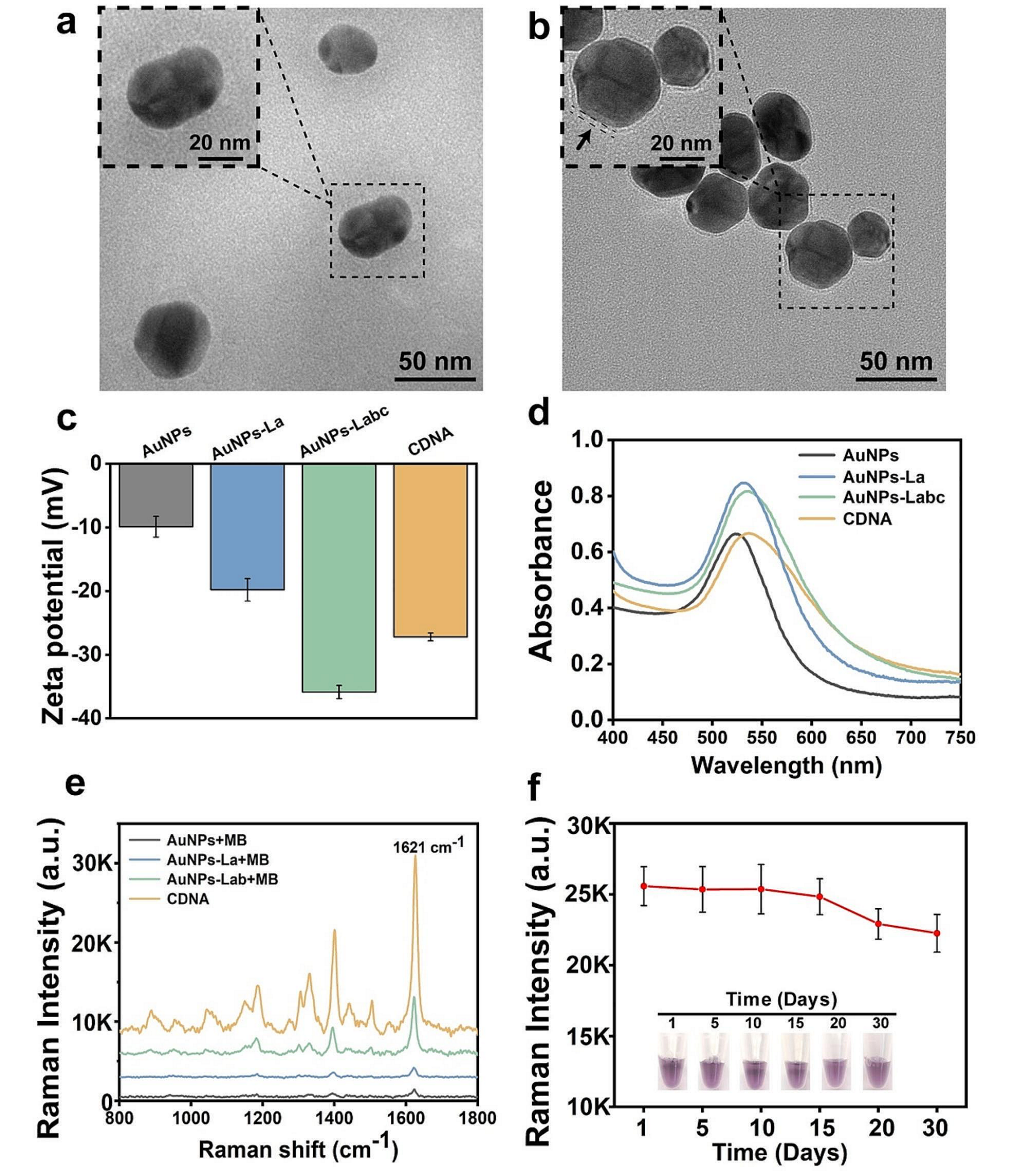
Characterization of the synthesized CDNA SERS tags. **a-b** Representative TEM images of AuNPs (a) and CDNA (b) (scale bars : 100 nm; scale bar in magnified image: 20 nm); **c** Zeta potentials of AuNPs, AuNPs-La, AuNPs-Labc and CDNA; **d** UV–vis spectra of AuNPs, AuNPs-La, AuNPs-Labc and CDNA; **e** SERS spectra of AuNPs + MB, AuNPs-La + MB, AuNPs-Lab + MB and CDNA; **f** The Raman intensity and color of CDNA after storage at 4 °C for 1, 5, 10, 15, 20 and 30 days

To characterize the assembly of the CDNA tags, transmission electron microscopy (TEM) was employed to determine the morphology of CDNA tags (Fig. 2a and b). As shown in Fig. 2b, distinct from the spherical and dispersed nature of AuNPs, the CDNA tags displayed a clear DNA/MB layer, indicating successful preparation of CDNA tags. Dynamic light scattering (DLS) measurements further revealed sizes of 45 nm (AuNP), 55 nm (AuNP-La), 75 nm (AuNP-Labc), and 78 nm (CDNA), which aligned with TEM observations (Supplementary Fig. S3). Zeta potential measurements and UV-Vis spectroscopy were adopted to further corroborate the features of the CDNA tags. Due to the negative charge of DNA, the AuNP-Labc had the lowest zeta potential (-35.87 mV) compared to naked AuNPs (-9.9 mV) and AuNP-La (-19.8 mV). The zeta potential of CDNA increased to -27.2 mV after loading with MB (Fig. 2c). A 3-nm UV-vis redshift was noted for CDNA (538 nm) in comparison to AuNPs (523 nm) and AuNP-Labc (535 nm) (Fig. 2d). These findings demonstrated that the three DNA strands and MB were successfully integrated onto the AuNP surface during in situ assembly.

To ascertain the efficacy of the CDNA tags, we performed Raman measurements on gold nanoparticles with unmodified amplified strands (AuNPs + MB), AuNPs-La + MB, AuNPs-Lab + MB and AuNPs-Labc@MB. The Raman intensity of the gold nanoparticles with amplified chains was significantly higher than that of nanoparticles without amplified chains, as evidenced by a clear peak at 1621 cm^− 1^ (Fig. 2e). The Raman intensities of AuNPs + MB and AuNPs-Labc@MB were further quantified, as shown in Supplementary Fig. S4. Figure 2f illustrates the exceptional stability of CDNA tags by showing the unchanged pink color of the tags even after a 30-day of storage at 4 °C, and the consistent SERS signal over the same duration. In addition, the CDNA tags maintained stability across various salt solutions (Supplementary Fig. S5). Overall, these findings show that the CDNA tags were successfully manufactured and had robust SERS signals and high stability.

### Feasibility of the biosensor

**Fig. 3:**
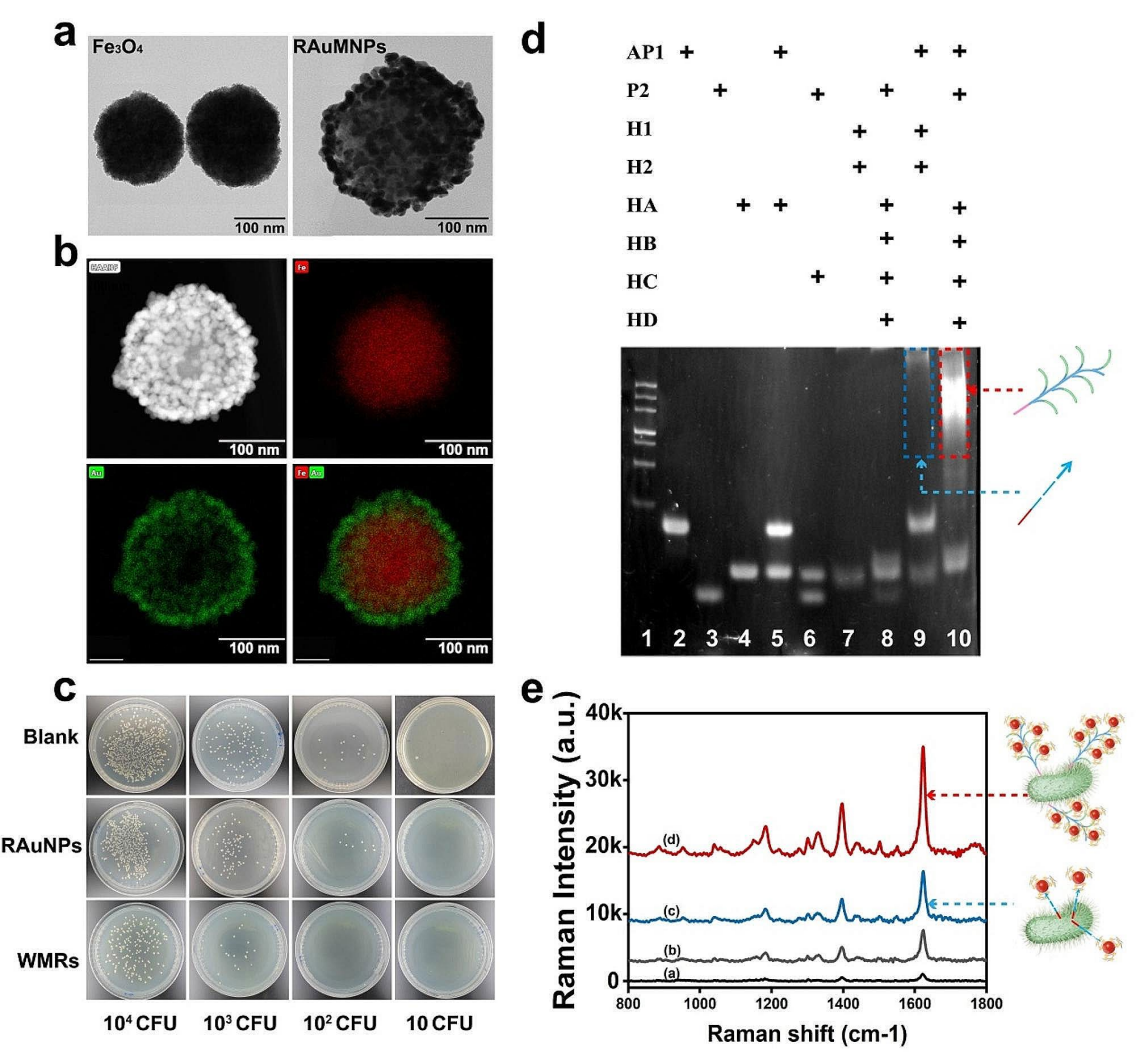
Evaluation of biosensor feasibility. **a** TEM of Fe_3_O_4_ MNPs and RAuMNPs; **b** EDS mapping results of RAuMNPs; **c** The number of *E. coli* O157:H7 in the supernatant after WMR capture; **d** PAGE analysis of the feasibility of cPER. L1: DNA marker; L2: AP1; L3: P2; L4: HA; L5: AP1 + HA; L6: P2 + HC; L7: H1 + H2; L8: AP1 + P2 + HA + HB + HC + HD; L9: AP1 + H1 + H2 L10: AP1 + P2 + HA + HB + HC + HD; **e** Raman intensity of the detection of *E. coli* O157:H7 with cPER and PER.

To verify the feasibility of this platform, we investigated the capture efficiency of WMRs. The WMRs were prepared following the methodology delineated in our prior publication [40] (as illustrated in Fig. 1c). Fe_3_O_4_ and WMRs with characteristic circular shapes were observed by transmission electron microscopy (TEM) (Fig. 3a-b). Upon the integration of the Au NP shell with Fe_3_O_4_, the diameter of these MNPs expanded from 200 to 240 nm. Through energy dispersive X-ray spectroscopy (EDS), the compositional integrity of the resultant RAuMNPs was assessed. Au was evenly distributed over the Fe core surface (Fig. 3b), indicating the effective synthesis of RAuMNPs with a core-shell architecture. The zeta potential shift from − 22.27 mV for Fe3O4@Au-MUA to -11.49 mV upon WGA modification implied the effective synthesis of WMRs (Fig. S6). The capture efficiency of WMRs was substantiated by mixing them with different concentrations (10–10^4^ CFU) of *E. coli* O157:H7. After WMRs were isolated, the number of leftover bacteria significantly decreased, whereas the RAuMNPs demonstrated no affinity for the target bacteria (Fig. 3c), indicating the robust capture effectiveness of WMRs specific to *E. coli* O157:H7.

The reliability of cPER was corroborated using PAGE experiments. As shown in Fig. 3d, in the presence of all substrates, a trapezoidal band with diminished gel mobility was observed (lane 10), indicating the formation of high molecular weight dendritic DNA nanostructures. The intensified band in lane 10 compared to lane 9 (linear DNA product) suggested that more DNA product is synthesized by dendritic PER over the linear PER. To demonstrate the signal amplification afforded by conjugated cPER, we first functionalized Linkers La and Lb with AuNPs as per the proposed protocol. Subsequently, the aptamer was hybridized to Lb, and MB was incorporated into the Aptamer-Tag (Apt-Tag). This Apt-Tag was then employed to label samples incubated with and without 5 × 10^5^* E. coli* O157:H7 followed by SERS analysis. The utilization of Apt-Tag for *E. coli* O157:H7 detection yielded only a marginal signal. When juxtaposed with the signals obtained via PER (c) and cPER (d), it was discernibly comparable to the signal from the control sample without *E. coli* O157:H7. However, with the introduction of cPER, a prominent signal (d) emerged, which was 2.2-fold more intense than that from conventional PER (c) and 4-fold stronger than that without any PER (b) (Fig. 3e). This clearly underscores the robustness and effectiveness of the constructed platform.

### Performance of the CDNA-based SERS platform

**Fig. 4:**
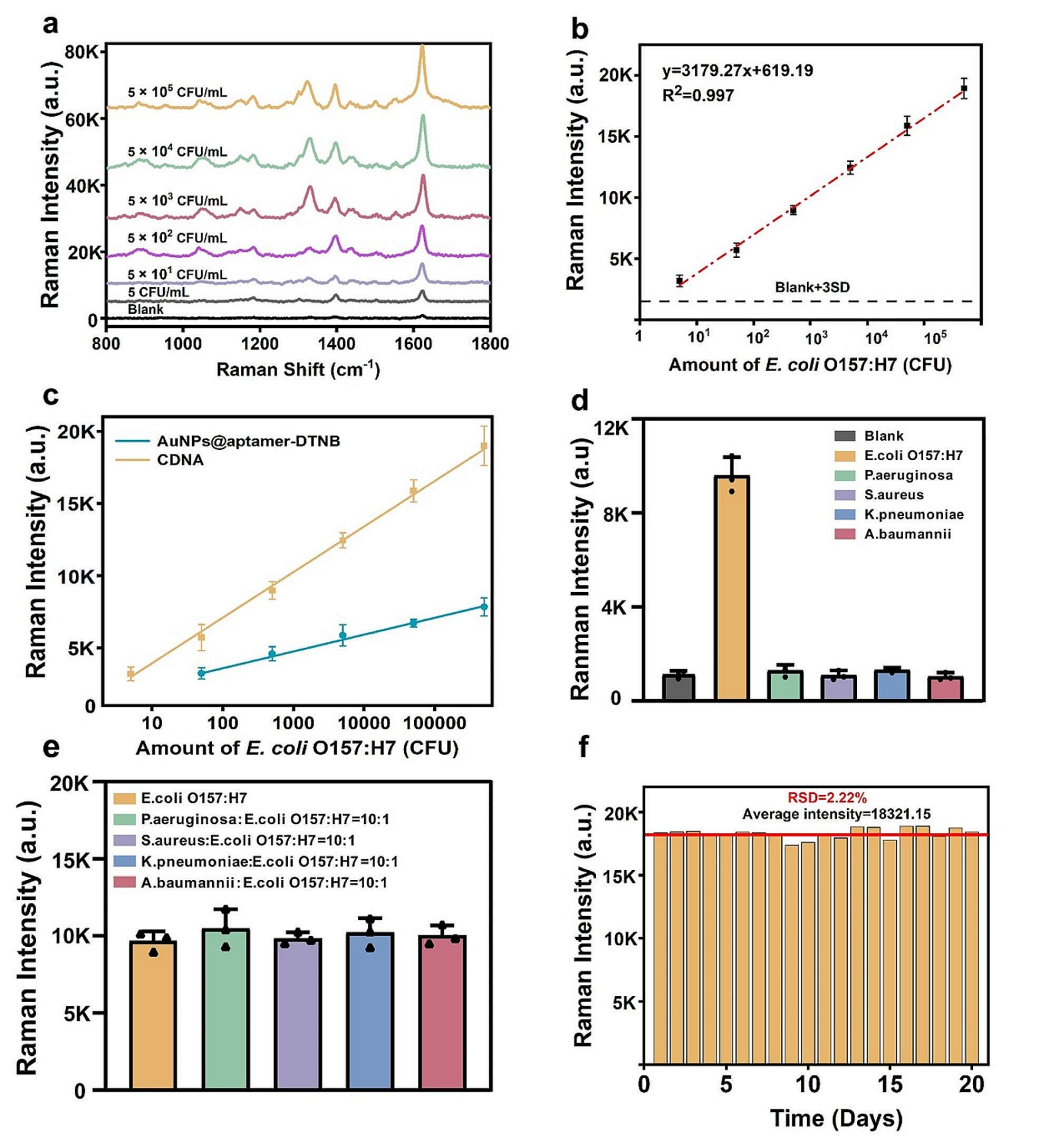
**a** Raman spectra of *E. coli* O157:H7 at different concentrations (5 ~ 5 × 10^5^ CFU mL^− 1^); **b** The linear relationship between *E. coli* O157:H7 concentration and Raman intensity at 1621 cm^− 1^; **c** Linear analysis of *E. coli* O157:H7 detection by traditional SERS tag (blue) and CDNA SERS tags (orange); **d** Specificity evaluation of the SERS platform, only *E. coli* O157:H7 generated obvious Raman signal; **e** SERS intensity between *E. coli* O157:H7 and other complicated bacteria samples, the amount of other pathogens was ten times that of *E. coli* O157:H7; **f** The Raman intensity of 20 randomly selected SERS spectra acquired from the measurements for 5 × 10^5^ CFU/mL of *E. coli* O157:H7.

To optimize the assay’s performance, several experimental parameters, including the concentration of Bst DNA polymerase, incubation time, and concentrations of Mg^2+^ and MB, were fine-tuned (Supplementary Fig. S7). Subsequently, different concentrations of target bacteria were used to test the detection sensitivity of the constructed platform. The concentration of *E. coli* O157:H7 was determined by the plate counting method (Supplementary Fig. S8). With increasing *E. coli* O157:H7 concentrations, a consistent rise in SERS intensity was observed (Fig. 4a). The linear connection was established using the typical Raman spectra of SERS tags at 1621 cm^− 1^. The signal indicated good linearity of *E. coli* O157:H7 concentrations ranging from 5 CFU/mL to 5 × 10^5^ CFU/mL, with a limit of detection (LOD) of 1.91 cells/mL (R^2^ = 0.997) (Fig. 4b). Notably, the CDNA-based SERS platform exhibited superior sensitivity when comapared with conventional SERS tags (Fig. 4c). Furthermore, when compared to other SERS tags for detecting *E. coli* O157:H7, our proposed platform demonstrated superior performance (Table 1) [37–41].

**Table 1:** Comparison with other SERS tags for *E. coli* O157:H7 detection

SERS tags	Total assay time (h)	LOD (CFU mL^-1^)	linear response (CFU mL^-1^)	References
ALP-Au-NRs	> 3	10	1.7 × 10^1^ ~ 1.7 × 10^6^	Bozkurt. 2018 [41]
TBDP@Au	2	10	10^1^ ~ 10^5^	Yang. 2022 [40]
GNS@4-MBA-Apt NPs	2.5	3.46	3.2 × 10^1^ ~ 3.2 × 10^7^	Ye. 2022 [42]
4ATP/Ag-pSi	> 3	3	10^1^ ~ 10^5^	Muthukumar.2023 [43]
4-MPBA@AuNPs)	> 10	1.35	10^2^ ~ 10^8^	Yang. 2022 [44]
Au@MMSPM	2	2.2	10^1^ ~ 10^6^	Wang.2023 [45]
CDNA	2	1.91	5 ~ 5 × 10^5^	Our work

The specificity of the developed biosensor was assessed against other pathogenic species (5 × 10^3^ cells/mL), including *P.aeruginosa*, *S.aureus*, *K. pneumoniae*, and *A. baumannii*. As exhibited as in Fig. 4d, the target bacteria *E. coli* O157:H7 showed a high intensity at 1621 cm^− 1^, which was over 9-fold higher than that of other nontarget groups. Mixtures of *E. coli* O157:H7 and other bacteria at a 1:10 molar ratio were used to further explore the specificity of this platform in complicated samples. There was no substantial variation in SERS intensity between *E. coli* O157:H7 and other complicated bacterial samples (Fig. 4e). The good stability and reproducibility of the proposed CDNA-based SERS platform was verified through 20 random measurements (Supplementary Fig. S9), and the RSD of the 20 random Raman intensities was 2.22% (Fig. 4f).

### Measurement of *E. coli* O157:H7 in real samples

**Fig. 5:**
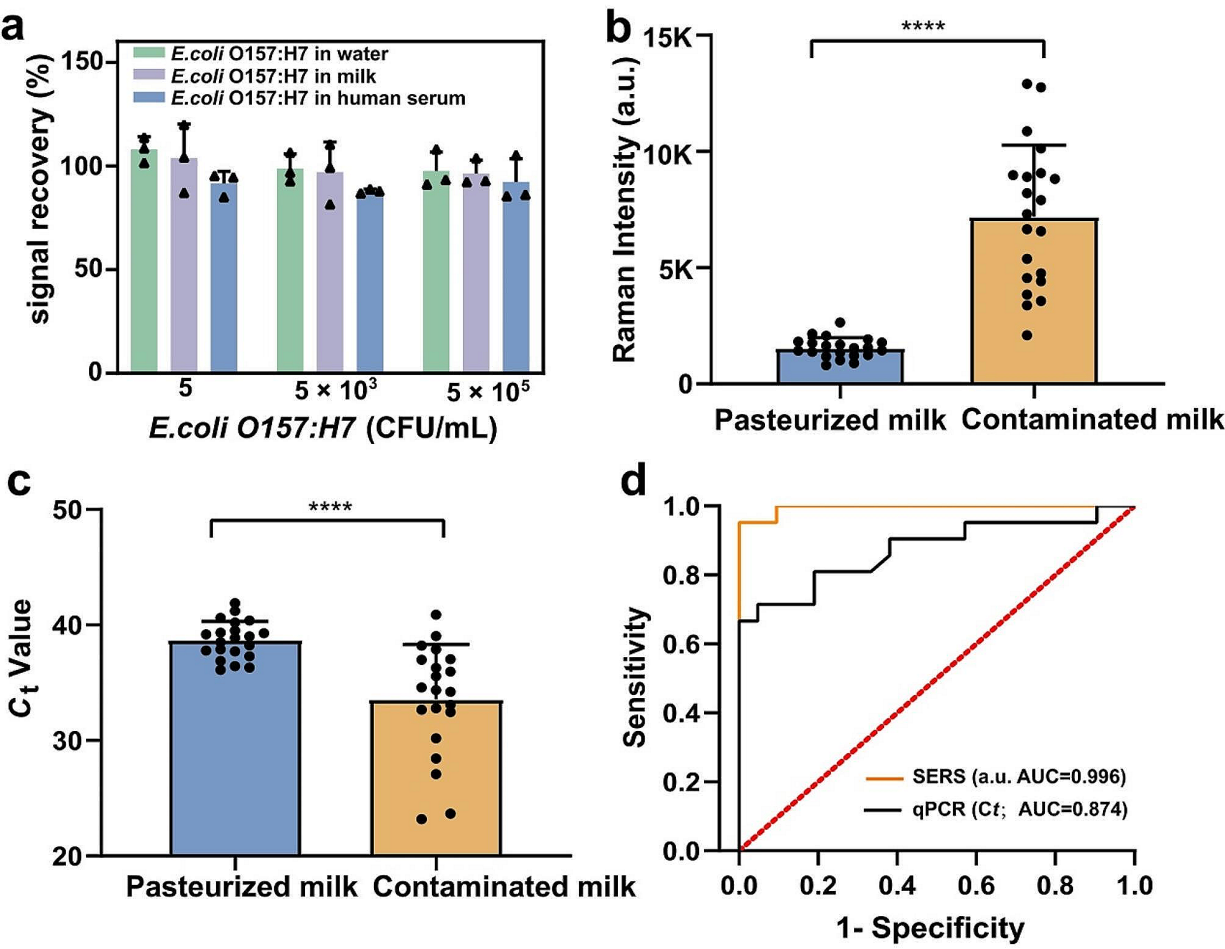
Measurement of *E. coli* O157:H7 in real samples. **a** Raman intensity of pasteurized milk versus contaminated milk; **b** Raman signal recovery of *E. coli* O157:H7 spiked in water, milk and human serum; **c** Ct value for genomic DNA of *E. coli* O157:H7 in pasteurized milk versus contaminated milk. (paired two-tailed Student’s t test, ****P < 0.0001); **d** ROC curve analysis of the CDNA-based SERS platform and qPCR.

Three sample types were selected to validate the accuracy of the CDNA-based SERS platform in real samples. As shown in Fig. 5a, the signals from spiked 5 × 10^5^ and 5 × 10^3^ CFU/mL of *E. coli* O157:H7 were recovered from 87.86 to 98.72% in three real samples. Even with a spike of just 5 CFU/mL *E. coli* O157:H7, recovery rates were recorded at 107.97% in water, 91.62% in milk, and 103.77% in human serum.

To underscore the platform’s practical applicability, we assembled a cohort of real samples consisting of pasteurized milk samples (*n* = 20) and contaminated milk samples (*n* = 20). As shown in Fig. 5b, the SERS intensity of *E. coli* O157:H7 was able to differentiate between contaminated milk and pasteurized milk, with the former exhibiting greater intensity. While qPCR measurements also identified differential *E. coli* O157:H7 signals between pasteurized milk samples and infected milk samples, there was an observable signal overlap in the signal percentages between the groups (Fig. 5c). Moreover, our proposed platform achieved an AUC of 0.996 (sensitivity: 95%, specificity: 100%) in contaminated milk samples, outperforming qPCR (AUC of 0.874, with both sensitivity and specificity at 81%) (Fig. 5d). The CDNA-based SERS platform has higher sensitivity than qPCR, possibly due to the following reason: sample preparation process for qPCR, such as nucleic acid extraction, might induce false negatives in low-load *E. coli* O157:H7 samples, whereas our nucleic acid-free extraction SERS platform, by directly detecting a cascade of amplified signals, potentially circumvents this issue.

### Measurement of ***E. coli*** O157:H7 in mouse serum

**Fig. 6:**
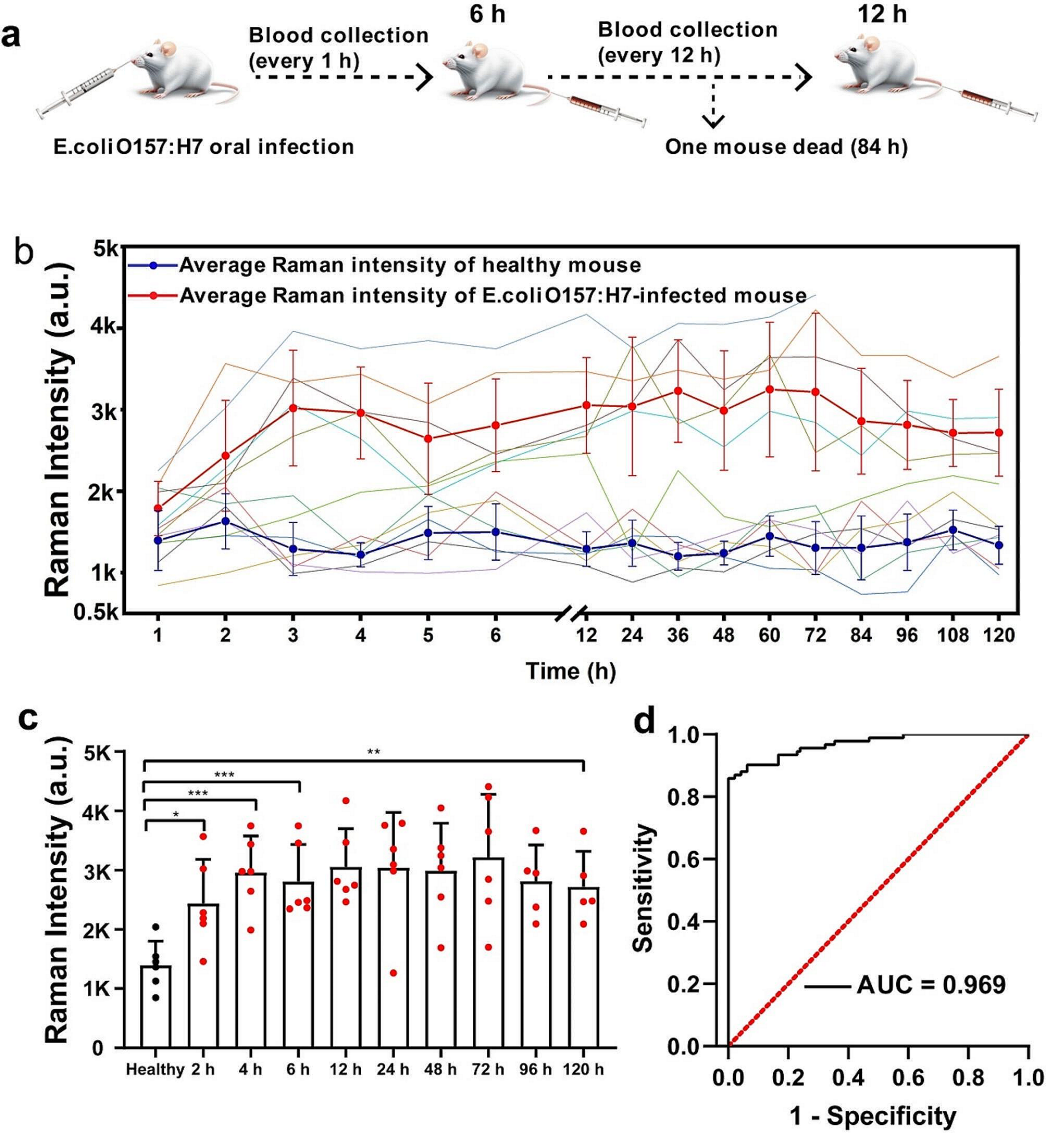
Measurement of *E. coli* O157:H7 in mouse serum. **a** Schematic representation of the methodology employed to establish an *E. coli* O157:H7-infected mouse model and subsequent blood collection; **b** Raman intensity profiles of six mice infected with *E. coli* O157:H7 and six healthy mice (represented by the broken line) are shown, along with the average trace (indicated by the red and black lines). The error bars represent standard deviations; **c** Raman intensity of *E. coli* O157:H7 expressed in serum of infected mice and healthy mice at the indicated times (paired two-tailed Student’s test, *P < 0.05, **P < 0.01, ***P < 0.001); **d** ROC curve analysis was performed to compare healthy mice (*n* = 6) with *E. coli* O157:H7-infected mice (*n* = 6)

Given the excellent performance of our proposed platform in complex samples, we extended its application to noninvasive monitoring of infection in vivo using mouse models after studying the stability and biocompatibility of WMRs (Fig. S10 and S11). We orally administered *E. coli O157:H7* preparations to 8 to 10 -week-old mice and subsequently monitored the presence of *E. coli O157:H7* presence in their serum (Fig. 6a). Our platform detected *E. coli* O157:H7 in each mouse hourly for the initial 6 h, followed by 12-hour intervals until 120 h. Clinical symptoms appeared 1 h post-administration of the *E. coli* O157:H7 preparation. Figure 6b and c illustrate the in vivo proliferation of *E. coli* O157:H7 in the infected mice, as evidenced by a marked increase in SERS intensity compared to uninfected mice. A minor decline in SERS intensity from 3 to 5 h might be attributed to immune-mediated bacterial clearance, as demonstrated by the previous study from Shen et al. [42]. The capacity to detect *E. coli* O157:H7 in mouse serum enabled the early identification of infection. This platform had a high AUC of 0.969 (sensitivity: 90%, specificity: 94%) (Supporting information Table S2) for infection diagnostics, indicating its significant potential for early infection analysis (Fig. 6d).

## Conclusion

In conclusion, we developed a novel SERS biosensor that can detect low concentrations of *E. coli* O157:H7. cPER is activated by the Aptamer-primer binding to the target bacterium, resulting in the formation of branched DNA products. The branched DNA products then hybridize with the CDNA tags, which have a remarkable capacity for binding, good stability, and amplified SERS intensity. This platform’s excellent sensitivity was verified by detecting traces of target *E. coli* O157:H7 bacterium without cross-reaction with other bacteria. In addition, the efficacy of the developed platform in detecting *E. coli O157:H7* in infected mice highlighted its potential in food safety and early infection diagnosis. Despite its three-step procedure, the platform boasts notable benefits: it negates the need for nucleic acid extraction, offers adaptability for various bacterial detections by simply altering the aptamer, and remains cost-efficient at a mere $0.95 per test (Supplementary Table S3). Consequently, this methodology holds significant potential for broader bacterial detection applications.

### Electronic supplementary material

Below is the link to the electronic supplementary material.

Supplementary Material 1

## Data Availability

Not applicable.
