# Supplementary material for: Highly sensitive SERS platform for pathogen analysis by cyclic DNA nanostructure@AuNP tags and cascade primer exchange reaction
Source: J Nanobiotechnology. 2024 Feb 26;22:75. doi: 10.1186/s12951-024-02339-1 (PMC10895721; doi:10.1186/s12951-024-02339-1)
Supplement: Supplementary file 1 — Supplementary Material 1 [file 12951_2024_2339_MOESM1_ESM.docx]

**Supporting Information**

**Highly sensitive SERS platform for pathogen analysis by cyclic DNA nanostructure@AuNP tags and cascade primer exchange reaction**

*Yunju Xiao^a,1^, Shihua Luo^c,1^, Jiuxiang Qiu^b,d^, Ye Zhang^b^, Weijiang Liu^a^, Yunhu Zhao^a^, YiTong Zhu^b^, Yangxi Deng^a^, Mengdi Lu^a^, Suling Liu^a^, Yong Lin^a^, Aiwei Huang^a^, Wen Wang^b^*, Xuejiao Hu^a^*, Bing Gu^a^**

^a^ Division of Laboratory Medicine, Guangdong Provincial People's Hospital (Guangdong Academy of Medical Sciences), Southern Medical University, Guangzhou 510000, PR China

^b^ Department of Laboratory Medicine, Nanfang Hospital, Southern Medical University, Guangzhou 510515, PR China

^c^ Center for Clinical Laboratory Diagnosis and Research, The Affiliated Hospital of Youjiang Medical University for Nationalities, Baise 533000, Guangxi, People’s Republic of China.

^d^ Department of Laboratory Medicine, Guangzhou Eighth People’s Hospital, Guangzhou Medical University, Guangzhou, 510515, China

*Corresponding author at: Guangdong Provincial People's Hospital (Guangdong Academy of Medical Sciences), Southern Medical University, Guangzhou 510000, PR China. Email address: gubing@gdph.org.cn (B. Gu).

Guangdong Provincial People's Hospital (Guangdong Academy of Medical Sciences), Southern Medical University, Guangzhou 510000, PR China. E-mail address: [huxuejiao@gdph.org.cn](mailto:huxuejiao@gdph.org.cn) (X.Hu).

Department of Laboratory Medicine, Nanfang Hospital, Southern Medical University, Guangzhou 510515, PR China. E-mail address:[wen.wang@qmul.ac.uk](mailto:wen.wang@qmul.ac.uk) (W.Wang)

^1^ These authors contributed equally to this work.

**Experimental Methods**

**Materials and Reagents**

Methylene blue trihydrate (MB) was purchased from Macklin Biochemical Co.,Ltd. (Shanghai, China). HAuCl_4_•4H_2_O and wheat germ agglutinin and were purchased from Sinopharm Chemical Reagent Co. (Shanghai). The Aptamer and DNA used for the PER reaction were synthesized by Sangon Inc (Shanghai, China) (Table S1). Bst DNA Polymerase Large Fragment (8000 U/μL), 10 × ThermoPol Reaction Buffer and Magnesium Sulfate Solution (100 mM) were obtained from New England Biolabs, Inc (USA). dATP, dTTP and dCTP were purchased from Sangon Inc (Shanghai, China). The DL20 DNA marker, 6 × Loading Buffer and 4S Red Plus Nucleic Acid Stain were purchased from Takara (Dalian, China). SYBR Green qPCR mix was purchased from Vazyme Biotech Co., Ltd. (Nanjing, China). Bacterial Genomic DNA Extraction Kit (Tiangen Biochemical Technology Co., Ltd (Beijing, China). *E.coli* O157:H7 was provided by Zheng's group (Yin Hua), and *S. aureus*, *P. aeruginosa*, [*A. baumannii*](http://www.baidu.com/link?url=Xnl5bfstdJxif0lqceeK5l0k6TSHtHGjlYix_Ec_XHmgR9FAgAkR1cXRX1XAbhlZ) and *K. pneumonia* were collected from Laboratory Medicine, Guangdong Provincial People's Hospital.

**Instruments and measurements**

Fe_3_O_4_ MNPs and RAuMNPs were imaged using transmission electron microscopy (TEM) with an FEI Talos F200X microscope (FEI Ltd., USA). The Malvern Zetasizer Nano ZS90 (Marvin Panaco Ltd., UK) was utilized to assess the zeta potential and DLS of AuNPs in the aqueous solutions. A Thermo Fisher Scientific, USA's Multiskan GO Microplate Reader was used to conduct UV-vis absorption spectroscopy. A Renishaw inVia-Qontor Raman Microscope (Renishaw, UK) was used to gather SERS spectra. Using ABI QuantstudioTM DX real-time PCR detection devices, real-time PCR was carried out. By establishing consistent detection circumstances, SERS detection was standardized.

In this work, we used a 785nm laser with a power of 300 mw, and the transmittance power of the instrument was 60%. During the detection process, we chose a 1% attenuation power. Therefore, the laser power on the surface of the sample calculated as 300 mW × 60% × 1% =1.8 mW.

**WMRs preparation**

The raspberry-like Fe_3_O_4_@Au magnetic nanoparticles (RAuMNPs) were constructed based on our previous study [1]. First, 100 mg of the purchased Fe_3_O_4_ (10 mg mL^-1^ ) and 5 mL of PEI aqueous solution were added to 40 mL of deionized water sonicated 30 min. Forty milliliters mL of negatively charged AuNP seed (3 - 5 nm) solution was added to the above prepared PEI-coated Fe_3_O_4_ nanosheets (Fe_3_O_4_@PEI) and sonicated for 40 min, and the Fe_3_O_4_-Au seed was collected by fast magnetic separation and dispersed in 40 mL of water. AuNP seeds were grown in situ on Fe_3_O_4_-Au to create the Fe_3_O_4_-Au nanoshell. To summarize, 40 mL of aqueous hydroxylamine hydrochloride (0.5 mg mL^−1^) containing 1 wt% PVP and 100 µL of HAuCl_4_ (5 mM) were sonicated and 1 mL of Fe_3_O_4_-Au seed solution was injected into it. The resultant RAuMNPs were resuspended in 500 ml of deionized water for later usage after being subjected to a 15-minute reaction and two magnetic separation washes. The following was the construction of WGA-modified RAuMNPs: In summary, a MUA solution was used to carboxylate the RAuMNPs. To be exact, 6 μL of MUA solution (10 mM) and 2 mL of RAuMNPs (10 mg/mL) were combined, and the combination was sonicated for one hour at room temperature. After that, surface-carboxylated AuMNPs were resuspended in 500 μL of 0.1 M MES buffer (pH 5.5) after being further enriched using a magnet. After that, 5 μL EDC (100 mM) and 10 μL NHS (100 mM) were used to activate the carboxyl groups on the Au shell for 20 minutes. Subsequently, the RAuMNPs were activated for two hours at 37 °C with 5 μL WGA. Following magnetic separation, the final step was washing and storing the products at 4 °C.

**Modification of** **CDNA SERS tags**

AuNPs (45 nm) were synthesized according to a prior report [2]. One hundred milliliters of the above synthesized AuNPs was mixed with DNA La (5 mL, 100 mM) and freeze for 10 min at -80 °C. The resulting AuNPs-La were centrifuged, repeatedly washed with PBS, and dispersed in 100 ml PBS buffer. Then DNA strands Lb (5 mL, 100 mM) and Lc (5 mL, 100 mM) were added, and the mixture was reacted at 37 °C overnight with gentle shaking. The as-obtained AuNPs-Labc were centrifuged, washed with PBS, and dispersed in 100 ml PBS buffer. After that, molecular beacon (MB) (1 mL, 10^-3^ M) was incubated with AuNPs-Labc for 2h.

**Polyacrylamide Gel Electrophoresis (PAGE)**

To verify the HPER process, 8% native polyacrylamide gel electrophoresis (PAGE) was employed. For 45 minutes, the procedure was run in 0.5 × TBE buffer at 120 V. Following 4S Red Plus staining, the gel was examined using UV gel imaging equipment (Cambridge, UK).

**Detection of *E.coli* O157:H7 by the SERS platform**

The VITEK2 Compact Microbiology Analysis System for Identification was used to confirm the identity of the standard strains of *E. coli* O157:H7 after they had been grown overnight at 37 °C on either LB medium or Columbia blood agar (Supplementary Fig. S1). Cultures of *E. Coli* O157:H7 were diluted in 10 mM PBS (pH 7.4) to gradient concentrations(5, 5 × 10^1^, 5 × 10^2^, 5 × 10^3^, 5 × 10^4^, 5 × 10^5^ CFU mL^-1^). Ten milliliters of WMRs (10 mg mL^-1^) and a mixture of *E. Coli* O157:H7 at varying concentrations were shaken and incubated for 20 minutes in PBS buffer (pH 7.4, containing 1 mM Ca^2+^, 1 mM Mg^2+^ and 10 mM NaCl). After magnetic separation, the WMRs/bacteria complexes were cleaned with PBST to get rid of any remaining free bacteria. Reaction buffer 1×ThermoPol buffer, 1 mM MgCl_2_, 0.1 U μL^-1^ Bst DNA polymerase, and 0.1 mM dATP/dTTP/dGTP were used for PER. To initiate linear PER (LPER), which has the potential to create linear DNA products, the WMRs/bacteria complexes, 200 nM aptamerprimer1, 100 nM hairpin 1 (H1), and 100 nM hairpin 2 (H2), were introduced into the reaction solution. To activate the branching PER that might produce branched DNA nanostructures, the WMRs/bacteria complexes, 200 nM aptamer primer1, 100 nM primer 2, 100 nM hairpin A (HA), 100 nM hairpin B (HB), 100 nM hairpin C (HC), and 100 nM hairpin D (HD), were combined with the reaction buffer. After adding CDNA tags, the mixture was incubated for one hour. The compounds that were formed were washed with ddH_2_O that contained 0.05% Tween.

**Detection of *E.coli* O157:H7 by real-time PCR**

Using a bacterial genomic DNA extraction kit, the genomic DNA of *E. coli* O157:H7 was isolated. Using the *E. coli* O157:H7 Shiga toxin genes (stx1) as target sequences, the NCBI Primer system was used to generate primers. Next, using ABI QuantstudioTM DX real-time PCR detection devices, real-time PCR was carried out using 1 × SYBR green qPCR mix, 200 nM primer pair, and varying quantities of 5 μL genomic DNA in a 25 μL reaction mixture.

**Detection of *E.coli* O157:H7 in** **real samples**

A predetermined quantity of *S. aureus*, *P. aeruginosa*, *A. baumannii*, and *K. pneumonia*, or their combination, was spiked into drinking water, milk, and human blood, respectively, for specificity testing. In the validation test, 20 milk samples were randomly contaminated with varying amounts of *E. coli* O157:H7, ranging from 10 to 1 × 10^4^ CFU, and subsequently mixed with 20 pasteurized milk samples. *E. coli* O157:H7 was found in the aforementioned samples using SERS and real-time PCR.

**Establishment of a mouse infection model**

We obtained C57BL/6 mice from the Guangdong Medical Laboratory Animal Center. The Guangdong Provincial People's Hospital's Scientific Research Ethics Committee approved all animal studies, which were carried out in compliance with the National Regulation of China for the Care and Use of Laboratory Animals (Guangdong Academy of Medical Sciences). *E. coli* O157:H7 was cultured in LB medium and extracted after five minutes of centrifugation at 3000 rpm. Twelve mice, aged six to eight weeks, were randomly split into two groups at random. One group received an oral infection with 100 mL of *E. coli* O157:H7 solution (10^9^ CFU/mL), while the other group served as a control. Every hour of infection in the early stages (0 ~ 6 h), mouse blood samples (20 mL) were taken. Blood samples were taken every 12 hours for 120 hours after the first 12 hours. Heparin-containing tubes were used to collect whole blood samples, which were then kept for subsequent use at 4 °C. Centrifugation was used to extract serum for three minutes at 1000 rpm.

**Statistical analysis.**

At least three replications of each experiment and test were conducted. Student's t test was used to compare the data, which are represented as the mean ± s.d. For data analysis, Origin 8 and GraphPad Prism version 5.0 were utilized.

**Table S1. DNA sequences used in this study.**

| **Name** | **Length (Mer)** | **Sequence (5’-3’)** | **Modification** |
| --- | --- | --- | --- |
| La | 42 | GATACGAAGCTATCGCTTTCCACCAGGTCAGACGTTTTTTTT | 3`-SH C6 |
| Lb | 69 | GCGATAGCTTCGTATCCGTCTGACCTGGTGGTTTTTTTTAGCAAGCGCATCGTAAGCTAGTAGGTATGC |  |
| Lc | 52 | CCGGGCCCTAACCCTAACCGCTTACGATGCGCTTGCTTTTGCATACCTACTA |  |
| AptamerPrimer1 | 50 | CCGGACGCTTATGCCTTGCCATCTACAGAGCAGGTGTGACGGGGTTCCCC |  |
| Primer 2 | 30 | TCATCTTCTTCATCTTAAGAAGATGACAGG |  |
| Hairpin A | 42 | AAGATGAAGCCCGGTTTTCCGGGCTTCATCTTGGGGAACC | 3`Inverted dT |
| Hairpin B | 41 | AAGATGAAGCCCGGTTTTCCGGGCTTCATCTTCTTCATCTT | 3`Inverted dT |
| Hairpin C | 34 | TTAGGGCCCGGTTTTCCGGGCCCTAACCTGTCAT | 3`Inverted dT |
| Hairpin D | 34 | TTAGGGCCCGGTTTTCCGGGCCCTAACCCTAACC | 3`Inverted dT |
| Hairpin1 | 34 | TTAGGGCCCGGTTTTCCGGGCCCTAAGGGGAACG | 3`Inverted dT |
| Hairpin 2 | 34 | TTAGGGCCCGGTTTTCCGGGCCCTAACCCTAACC | 3`Inverted dT |
| stx1F | 26 | GTGGCATTAATACTGAATTGTCATCA | No |
| stx1R | 21 | GCGTAATCCCACGGACTCTTC | No |


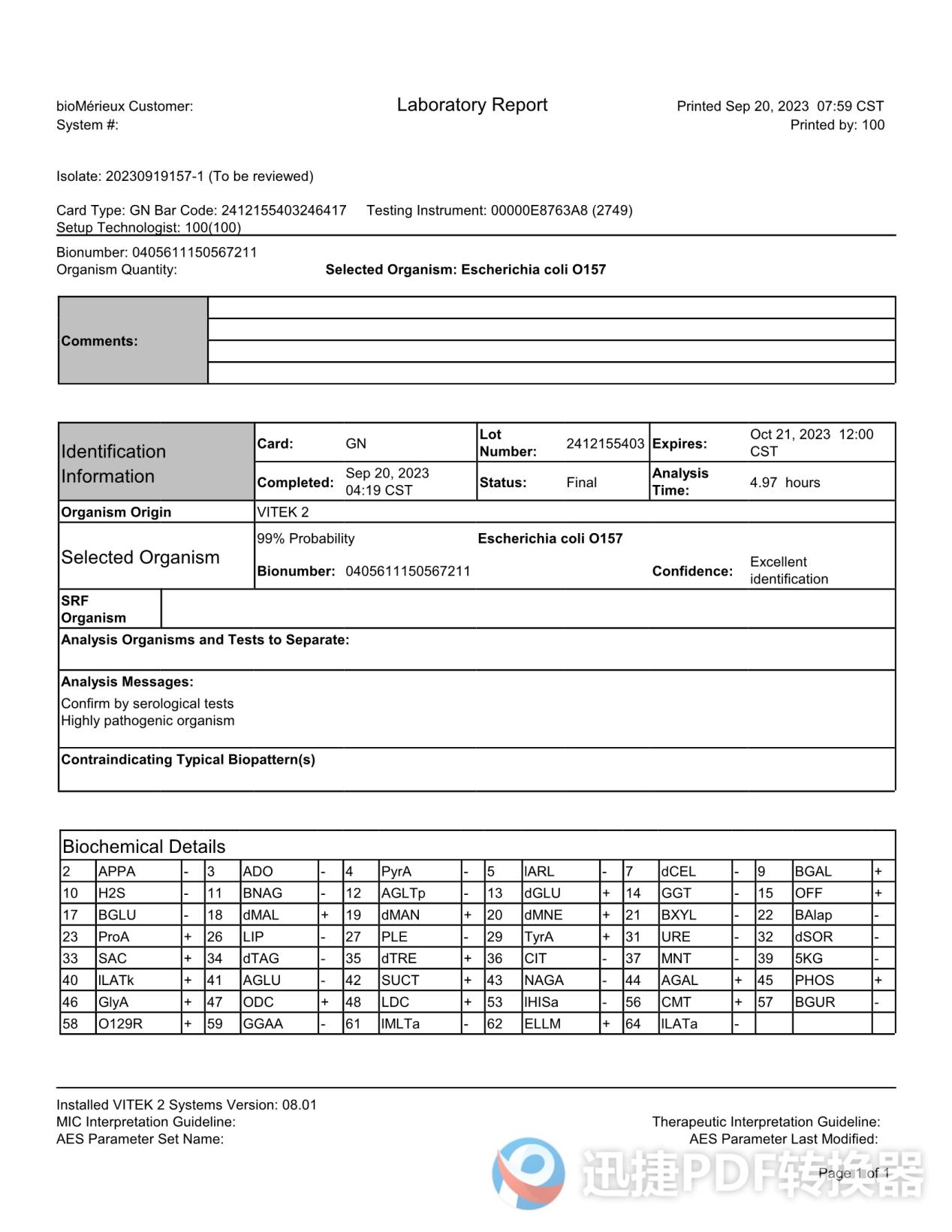


**Figure S1. Identification of *E. coli* O157:H7 by VITEK2 Compact Microbiology Analysis System.**

**
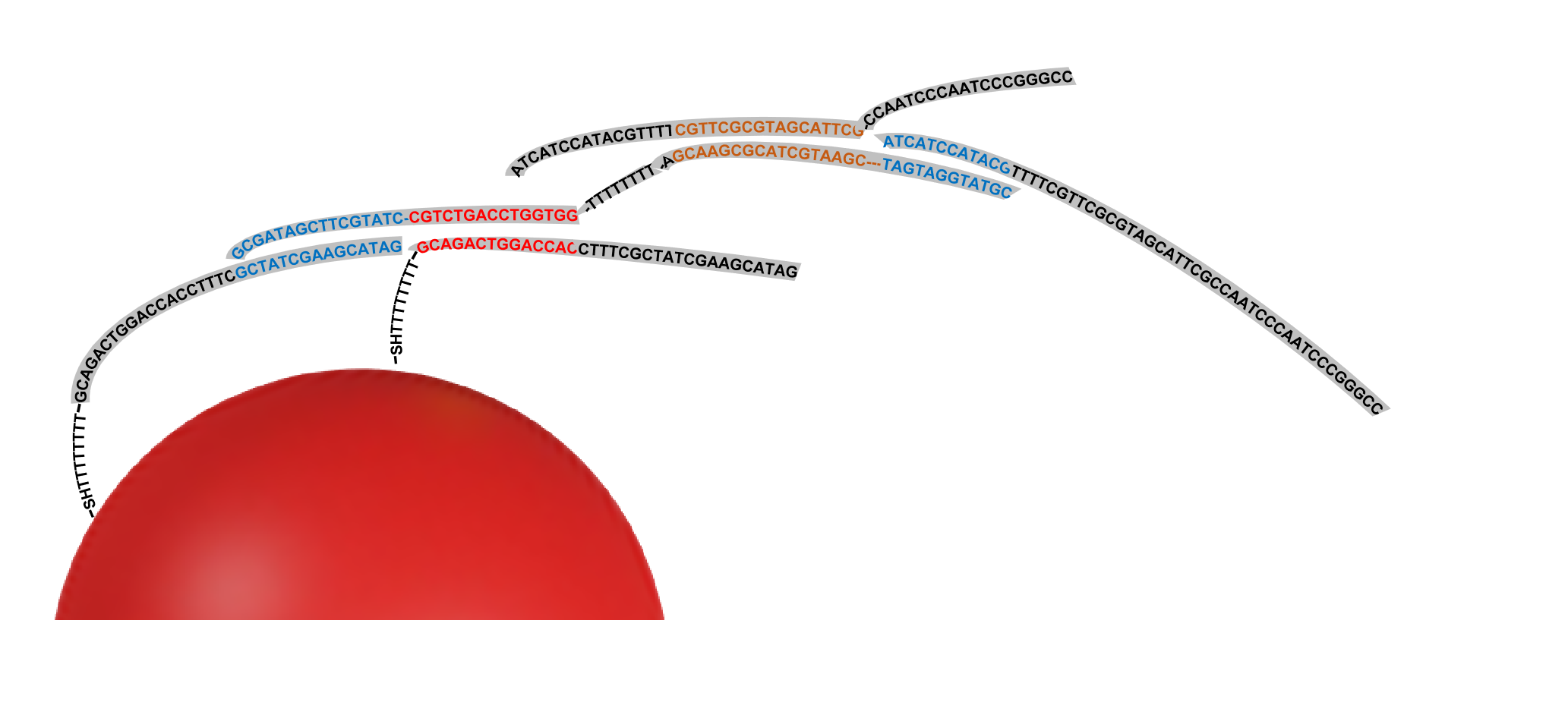
**

**Figure S2. Schematic illustration of the hybridization among La, Lb and Lc on AuNPs.**


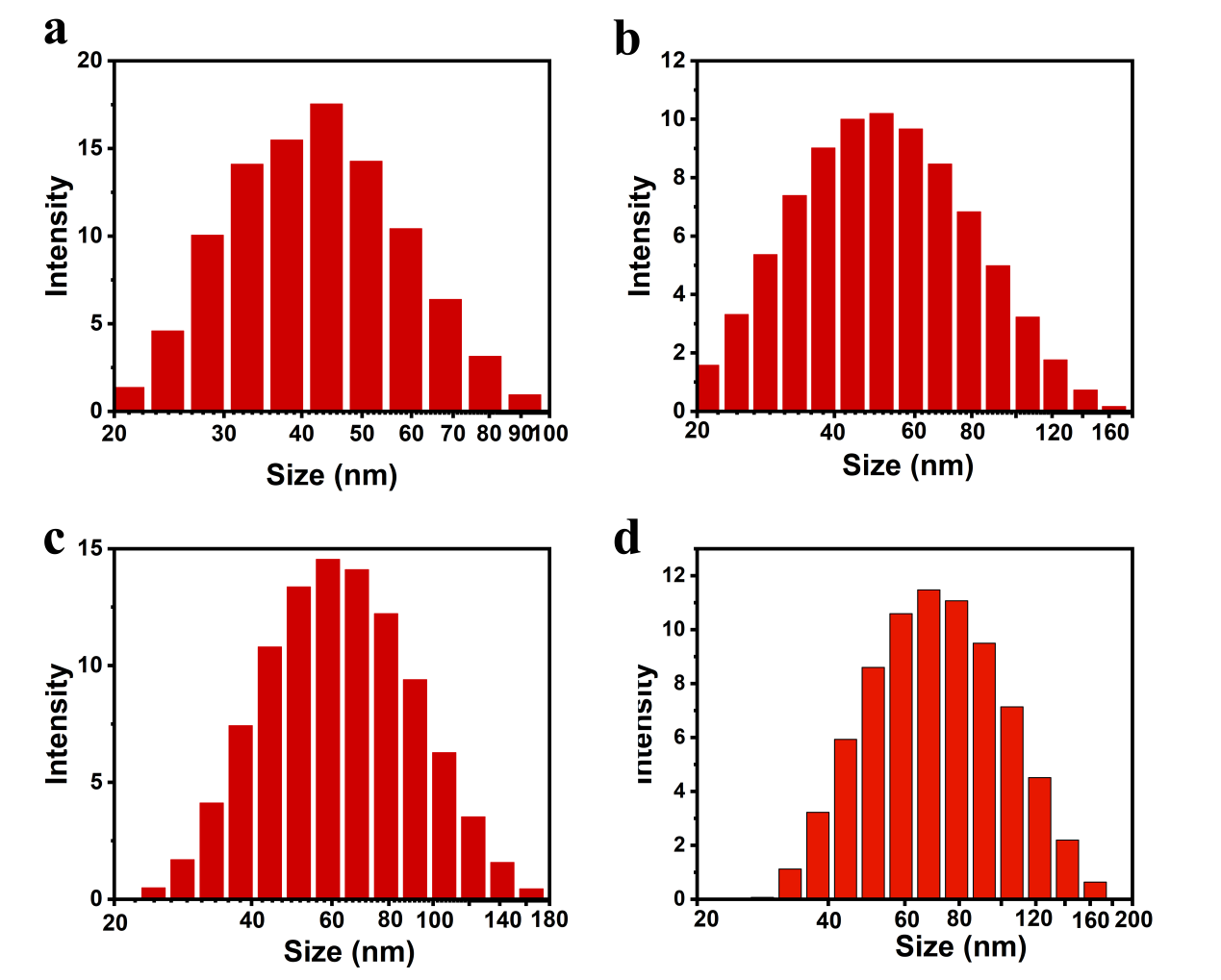


**Figure S3. DLS distributions of the bare AuNPs****(a),** **AuNP-La(b), AuNP-Labc(c) and CDNA tags(d).**


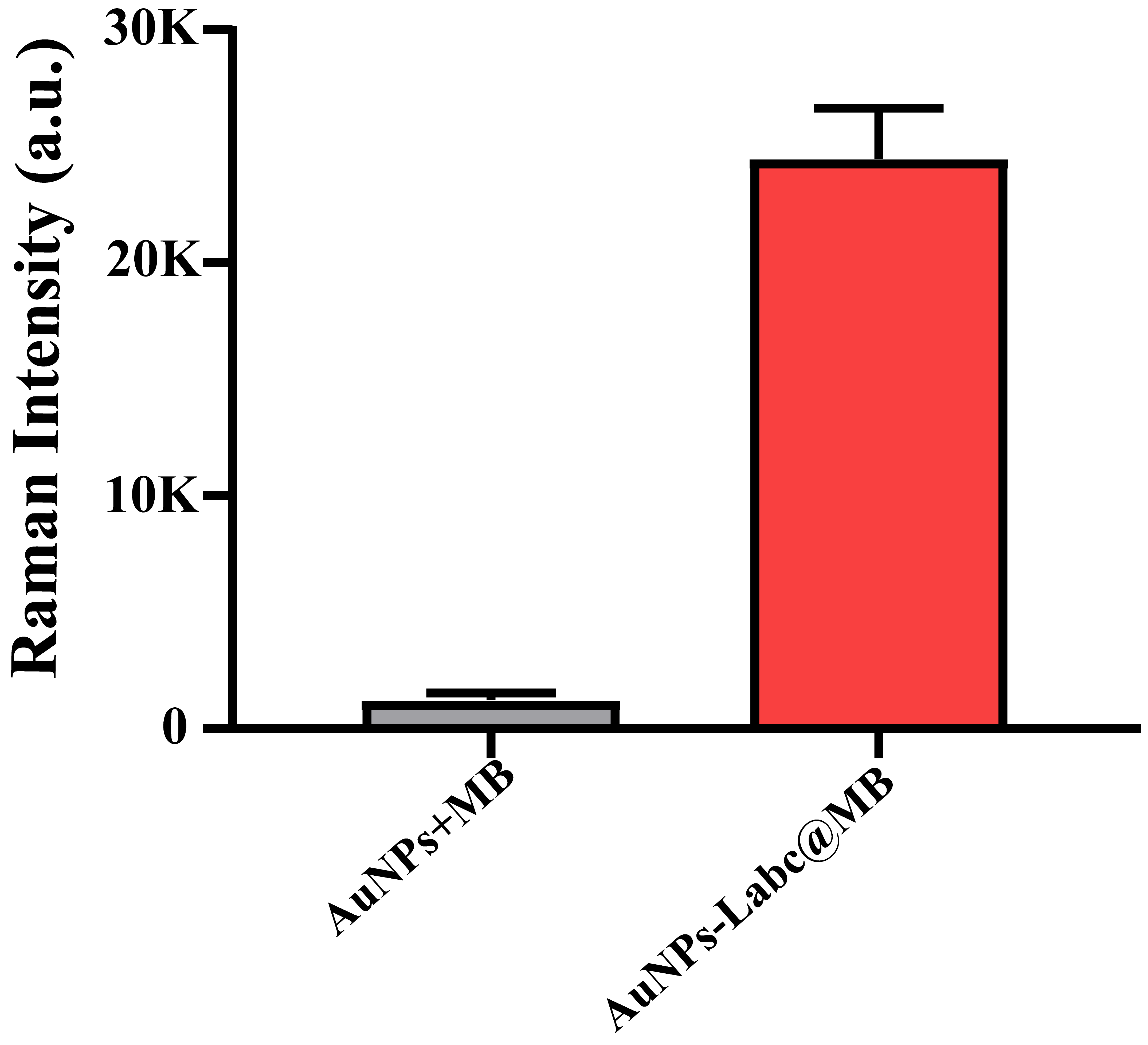


**Figure S4.** **Raman intensity of CDNA SERS tags**.

**
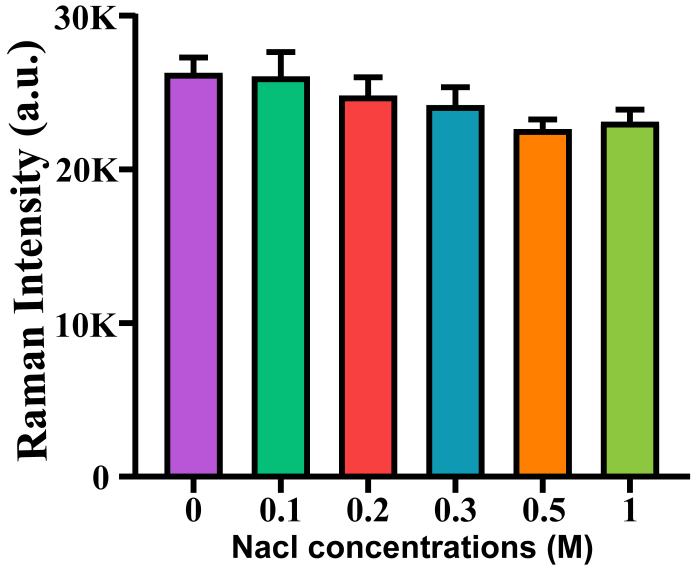
**

**Figure S5.** **The stability of the CDNA tags in different concentration of NaCl.**

**

**

**Figure S6. Zeta potentials of the Fe****_3_O_4_ nanoparticles, RAuMNPs, RAuMNPs-MUA and RAuMNPs-WGA(WMRs).**


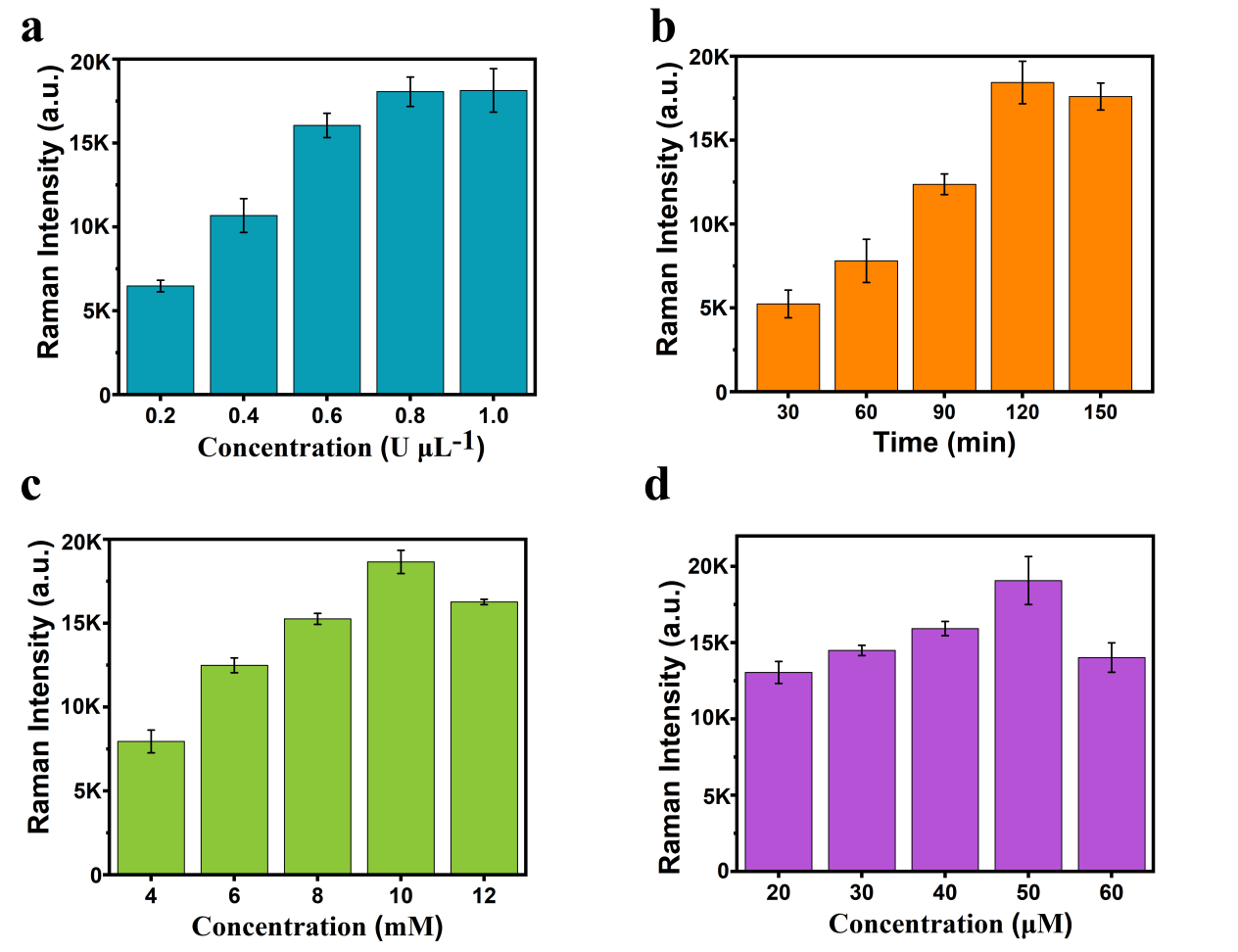


**Figure S7.** **Optimization of the detection conditions**. The effect of (a) the concentration of Bst DNA polymerase, (b) the incubation time, (c) the concentration of Mg^2+^ and (d) the concentration of MB. Data were expressed as the mean ± standard deviation, sample replicates n = 3.


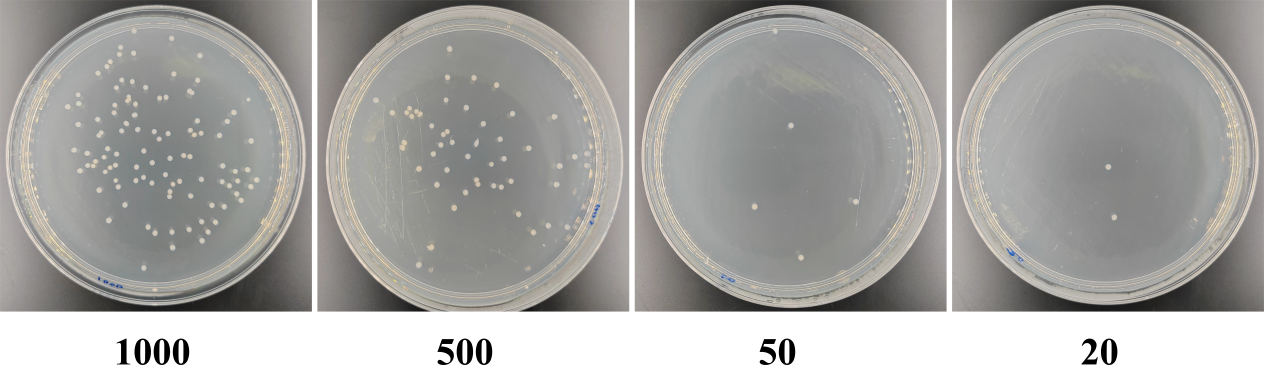


**Figure S8.** **The concentration of *E. coli* O157:H7 were verified by the** **plate counting method.** One hundred microliters of the bacterial samples (1000, 500, 50, 20 CFU/mL) was coated on LB plates.





**Figure S9. Repeatability and Stability of the SERS platform:** Twenty randomly selected SERS spectra acquired from the random measurements for 5 × 10^5^ CFU/mL *E. coli* O157:H7.


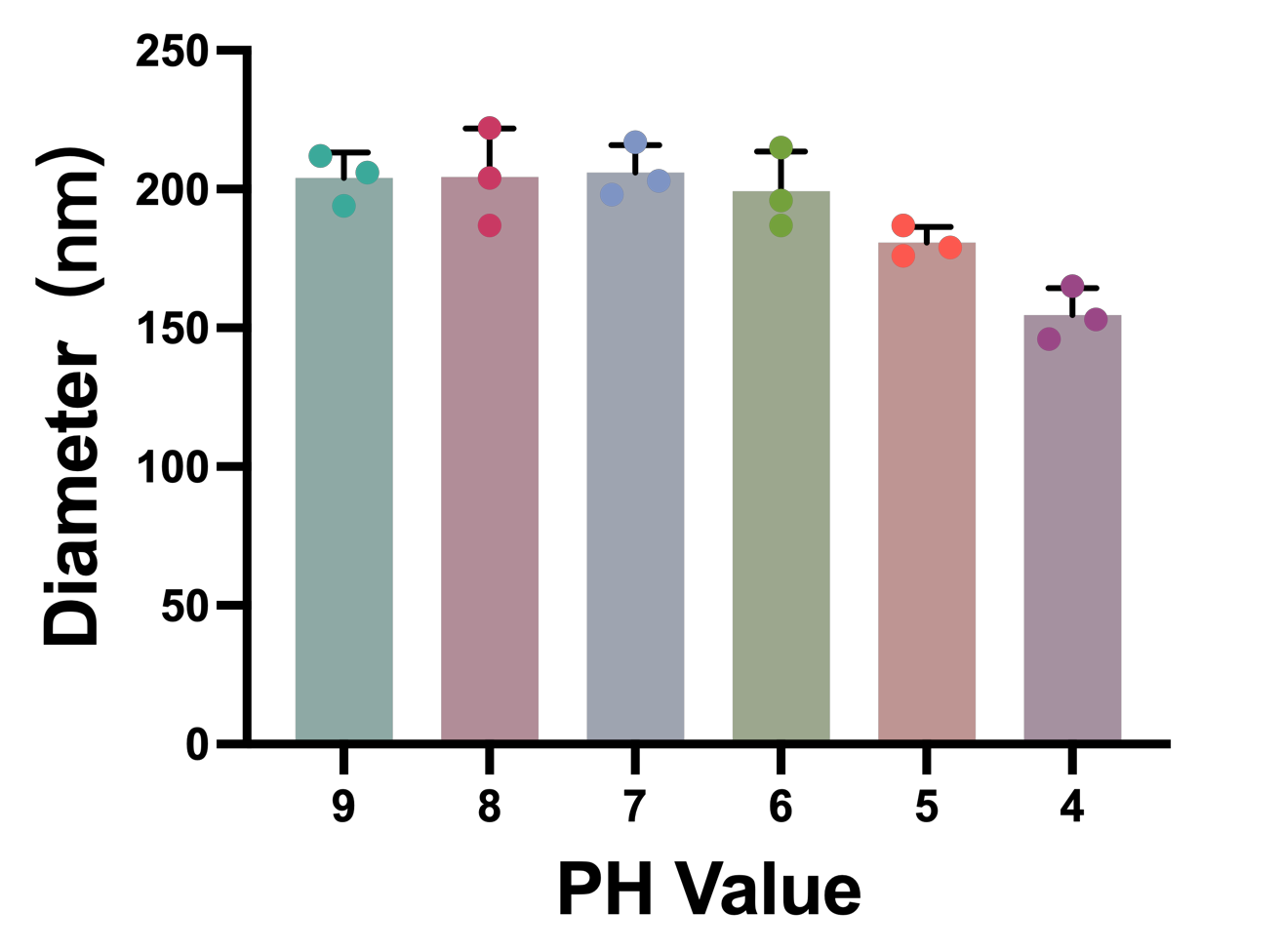


**Figure S10. Stability of WMRs in different pH solutions.**


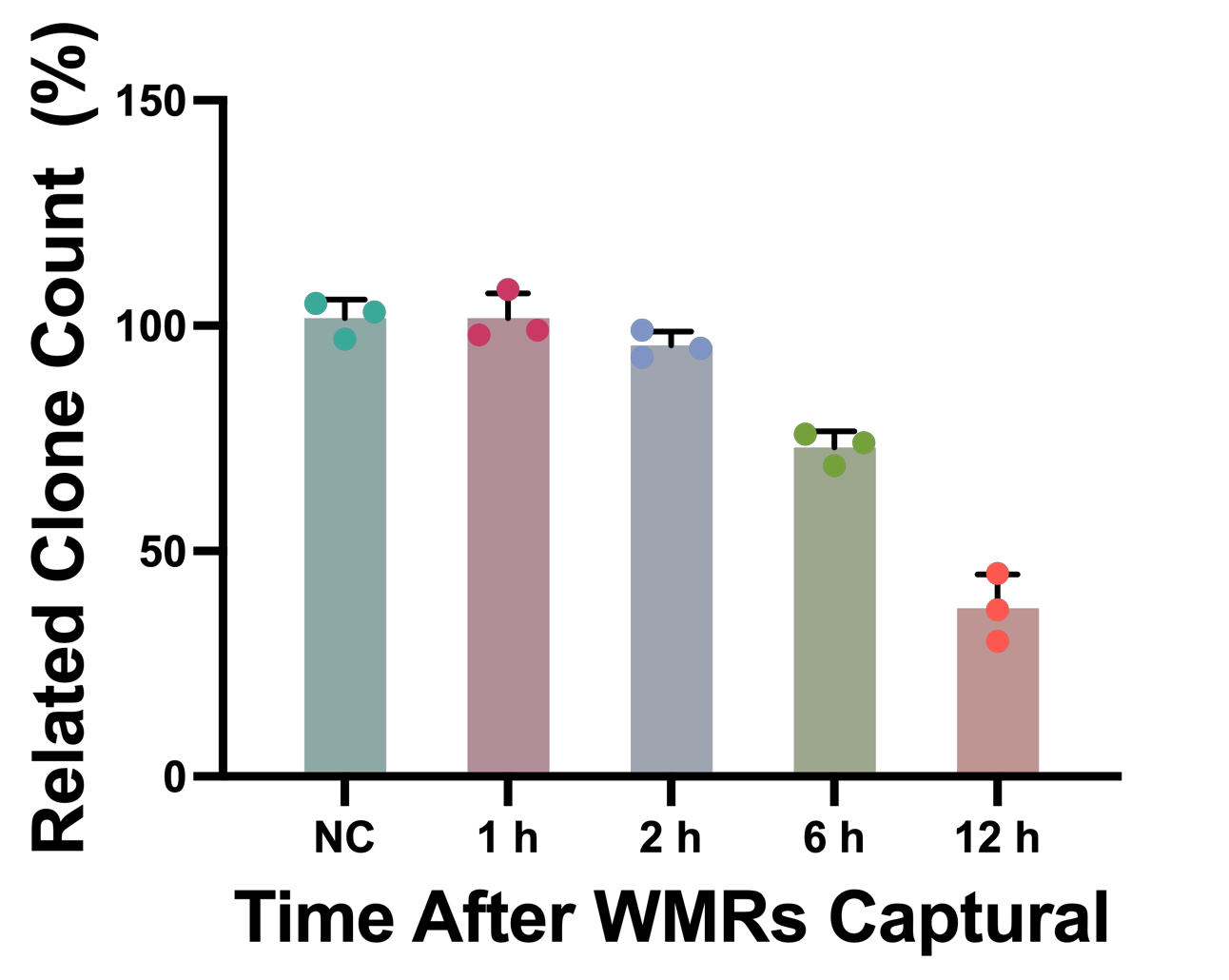


**Figure S11. Toxic study by clone counting.**

**Table S2 | Table associated with ROC curve analysis depicted in Fig.6D.**

| Parameter | AUC | Sensitivity | Specificity | 95%CI |
| --- | --- | --- | --- | --- |
| SERS/HPER | 0.969 | 0.90 | 0.94 | 0.947-0.991 |

**Table S3| Cost analysis**

| Step | Components | Amounts per 100 reactions | Costs per 100 reactions ($) |
| --- | --- | --- | --- |
| Preparation of RAuNPs-WGA | WGA | 100 mg | 7.47 |
|  | RAuMNPs | 10 mg | 3 |
|  | Reaction buffer | 100 mL | 0.74 |
| SERS tags | AuNPs | 16 mL | 0.12 |
|  | SH-aptamer | 400 mmol | 40 |
|  | MB | 10 mg | 2.14 |
| cPER | dNTP | 200 μmol | 8.5 |
|  | Bst DNA Polymerase | 1600 U | 14.5 |
|  | HA,HB,HC,HD,Primer2 | 2 mmol of each | 12.5 |
| SERS analysis | Si | 0.025 m^2^ | 6.57 |
|  | Reaction buffer | 1000 mL | < 0.1 |
| Total cost per 100 reacrtions | | | 95.54 |

References

1. W. Shen, C. Wang, X. Yang, C. Wang, Z. Zhou, X. Liu, R. Xiao, B. Gu, and S. Wang, Synthesis of raspberry-like nanogapped Fe3O4@Au nanocomposites for SERS-based lateral flow detection of multiple tumor biomarkers. J. Mater. Chem. C. 8 (2020) 12854-12864.
2. Xiao Y, Liu W, Zhang Y, Zheng S, Liao J, Shan H, Tian B, Wu T, Zhang L, Tu Z: Simple and rapid co-freezing construction of SERS signal probes for the sensitive detection of pathogens. Chem Eng J 2023, 466:143066.
